# Olfactory Reception of Host Alarm Pheromone Component by the Odorant-Binding Proteins in the Samurai Wasp, *Trissolcus japonicus* (Hymenoptera: Scelionidae)

**DOI:** 10.3389/fphys.2020.01058

**Published:** 2020-09-03

**Authors:** Jinping Zhang, Yongzhi Zhong, Rui Tang, K. B. Rebijith, Fengqi Li, Guohua Chen, Feng Zhang

**Affiliations:** ^1^MARA-CABI Joint Laboratory for Bio-safety, Institute of Plant Protection, Chinese Academy of Agricultural Sciences, Beijing, China; ^2^College of Plant Protection, Yunnan Agricultural University, Kunming, China; ^3^Institute of Plant Protection and Agro-products Safety, Anhui Academy of Agricultural Sciences, Hefei, China; ^4^Guangdong Key Laboratory of Animal Conservation and Resource Utilization, Guangdong Public Laboratory of Wild Animal Conservation and Utilization, Guangdong Institute of Applied Biological Resources, Guangdong Academy of Sciences, Guangzhou, China; ^5^Plant Health and Environment Laboratory, Ministry for Primary Industries, Auckland, New Zealand; ^6^Beijing Key Laboratory of Environment Friendly Management on Fruit Diseases and Pests in North China, Institute of Plant and Environment Protection, Beijing Academy of Agriculture and Forestry Sciences, Beijing, China; ^7^College of Agriculture and Ecological Engineering, Hexi University, Zhangye, China

**Keywords:** brown marmorated stink bug, egg parasitoid, insect olfaction, (*E*)-2-decenal, transcriptome

## Abstract

The samurai wasp, *Trissolcus japonicus*, is the predominant egg parasitoid of the brown marmorated stink bug, *Halyomorpha halys*, in its native range in China. (*E*)-2-Decenal is a major component of the alarm pheromone of *H. halys*, an important invasive insect pest with significant economic importance. *T*. *japonicus* can be strongly repelled by (*E*)-2-decenal, and thus its host location efficiency would be reduced in the field. Better understanding on the molecular basis of olfactory reception of this host alarm pheromone component by *T. japonicus* may provide opportunities to develop novel approaches to enhance biological control efficacy of the parasitoid against *H. halys*. We identified six Odorant Binding Proteins (OBPs) from *T. japonicus* by transcriptome sequencing, within which three classical OBPs were expressed in a heterologous expression system with *E. coli*, harvested, and then challenged with (*E*)-2-decenal in binding assay experiments. *Tjap*OBP2 showed the highest binding ability to (*E*)-2-decenal, compared to *Tjap*OBP1 and *Tjap*OBP3. Our results unambiguously suggest that *Tjap*OBP2 would play an important role in discriminating (*E*)-2-decenal and could be a possible target for further intervention in the parasitoid–host system.

## Introduction

Olfaction plays a crucial role in insects to sense biologically meaningful chemical signals in their surroundings for food, a mate, oviposition, prey, or enemies (Schneider, 1969; Bruyne and Baker, 2008; Conchou et al., 2019). Insect olfaction starts with reception of odorants at the periphery level during which a variety of olfactory proteins are involved in this early olfactory process, including odorant-binding proteins (OBPs), chemosensory proteins (CSPs), odorant-degrading enzymes (ODEs), odorant receptors (ORs), ionotropic receptors (IRs), and sensory neuron membrane proteins (SNMPs) (Vogt and Riddiford, 1981; Steinbrecht, 1998; Leal, 2013; Pelosi et al., 2018). Despite a variety of physiological roles, the OBPs are essential for a functional olfactory system in insects, acting as carriers of odorants and liaisons between the external environment and ORs (Steinbrecht, 1998; Leal, 2005, 2013; Pelosi et al., 2018). Since its first detection in the giant moth *Antheraea polyphemus* in 1981 (Vogt and Riddiford, 1981), a large number of OBPs have been identified in different insect species, particularly phytophagous insect pests, social insects, and mosquitoes (Calvello et al., 2003; Zhou et al., 2004; Fan et al., 2011; McKenzie et al., 2014; Pelosi et al., 2018; Li et al., 2019), while there are only a few studies with parasitoids of insect pests (Vieira et al., 2012; Li et al., 2015; Zhou et al., 2015).

The brown marmorated stink bug (BMSB), *Halyomorpha halys* (Stål) (Hemiptera: Pentatomidae), native to East Asia (Hoebeke and Carter, 2003), has become a global invasive insect pest since its introduction into North America, Europe, and South America in 1990s, 2000s, and 2017, respectively (Haye and Weber, 2017; Valentine et al., 2017; Leskey and Nielsen, 2018). The BMSB has a broad host range with over 170 plants, many of which are economically important fruits, vegetables, and field crops (Lee et al., 2013; Leskey and Nielsen, 2018). In northern China, BMSB caused 50–80% losses in peach and pear production in the 1980s (Qin, 1990). In addition to its agricultural pest status, BMSB is regarded as an important nuisance pest as adults often overwinter in large aggregation populations in human-made structures and produce a pungent and unpleasant odor resulting from its defensive chemicals (Lee et al., 2013; Haye et al., 2015). Management of BMSB is still heavily relying on chemical pesticides, particularly broad-spectrum insecticides such as pyrethroids and neonicotinoids (Lee et al., 2013; Leskey and Nielsen, 2018). However, the extensive use of broad-spectrum insecticides may have adverse effects such as environmental pollution and residual problems for the health of farmers and consumers (Leong et al., 2020), increased chances of insecticide resistance (Haye et al., 2015), and outbreak of secondary pests (Leskey et al., 2012). To overcome these shortcomings of chemical control, it is very important to develop an alternative, safe, and eco-friendly method based on integrated pest management (IPM) practices, such as biological control, for sustainable management of BMSB.

The samurai wasp, *Trissolcus japonicus* (Ashmead) (Hymenoptera: Scelionidae) (syn. *T. halyomorphae* Yang; Talamas et al., 2013) is an important egg parasitoid of BMSB in its native range, causing high levels of parasitism up to 70% with significant control effects in the field in northern China (Yang et al., 2009; Zhang et al., 2017). Recent studies showed that adventive populations of *T. japonicus* were present in the United States (Talamas et al., 2015; Herlihy et al., 2016; Milnes et al., 2016; Hedstrom et al., 2017; Leskey and Nielsen, 2018), Switzerland (Stahl et al., 2018), Italy (Sabbatini Peverieri et al., 2018), and Canada (Abram et al., 2019). Although non-target risk of the samurai wasp would be a concern (Hedstrom et al., 2017; Botch and Delfosse, 2018; Haye et al., 2019; Milnes and Beers, 2019), the presence of *T. japonicus* together with endemic natural enemies might enhances the biological control of BMSB in North America and Europe (Leskey and Nielsen, 2018; Haye et al., 2019).

Our previous study showed that *T. japonicus* senses chemical volatiles released by BMSB adults, and the samurai wasp was attracted by n-tridecane while strongly repelled by (*E*)-2-decenal (Zhong et al., 2017). The strong repellence of (*E*)-2-decenal would reduce the efficiency of *T. japonicus* attacking BMSB. Furthermore, the alarm function of (*E*)-2-decenal and its olfactory perception by BMSB were characterized on behavioral and molecular levels (Zhong et al., 2018). However, the molecular mechanism underlying the olfactory perception of the alarm pheromone component in *T. japonicus* is still unknown. Thus, we carried out transcriptome sequencing to identify three classical ones from totally six OBPs in *T. japonicus* that are playing key roles in the process of olfaction. Our study also performed a competitive fluorescence-binding assay with the putative OBPs to elucidate the molecular mechanism underlying the recognition of (*E*)-2-decenal by *T. japonicus*. Thus, our work paves the way on the molecular basis of olfaction in *T. japonicus*, which further help in the inter-specific chemical communication between *T. japonicus* and BMSB.

## Materials and Methods

### Insects

*Trissolcus japonicus* was originally obtained from egg masses of *H. halys* collected from a peach orchard in Beijing, China (E116°12′41″; N40°02′06″) in June 2016 and used for the experiments after four generations of rearing in laboratory. *Halyomorpha halys* adults were continuously reared on a diet of pods of organic green beans (*Phaseolus vulgaris* L.) and cobs of corn (*Zea mays* L.) in rearing cages (60 × 60 × 60 cm). Eggs were collected daily and maintained in separate rearing cages until completion of the nymphal stage or provided to *T. japonicus* for the rearing of the parasitoid. Laboratory colonies of the samurai wasp were continuously reared with *H. halys* egg masses, maintained in transparent acrylic rearing cages (25 × 25 × 25 cm) and fed daily with a cotton wick saturated with 10% honey solution under laboratory condition of 25 ± 1°C, 65 ± 5% RH and 16 L: 8 D photoperiod (Zhong et al., 2017). Mated 3-day-old *T. japonicus* females were collected and used in all the experiments.

### RNA-Seq Construction and Sequencing

A total of 800 antennae, 400 heads (without antennae), or abdomen from *T. japonicus* females were grounded in TRIzol reagent (Invitrogen, Life Technologies, Carlsbad, CA, United States), and total RNA was obtained by the TaKaRa MiniBEST Universal RNA Extraction Kit (TaKaRa, Dalian, China). The RNA concentration and the ratio of OD260/OD280 were measured by NanoDrop. Data integrity of RNA was tested by Agilent 2100. The RNA samples (2.5 μg) extracted from *T. japonicus* tissues were sent to LC-Bio Co. (Hangzhou, China) for cDNA library construction and RNA-Sequencing (RNA-Seq). Enrichment and purification for mRNA were conducted by magnetic beads with Oligo (dT), then the fragmentation buffer was added to make the mRNA become a short segment. The mRNA was used to the template, and the first strand of cDNA was synthesized by a random primer of six bases. Then, the second strand of cDNA was synthesized by the DNA polymerase, and the double strands were purified by AMPure XP beads to obtain the finally library. The cDNA library with sufficient quality was sequenced by Illumina HiSeq^TM^ 2000 (Illumina, Inc., San Diego, CA, United States).

### Transcriptome Assembly and Annotation of Functional Genes

Clean reads were obtained after quality control by getting rid of the reads unconfirmed, with adapter and low quality. The assembly was conducted with the Trinity software (Haas et al., 2013) using the jellyfish kmer method (with default parameter settings). Gene annotations were performed using BLAST algorithm-based searches with an E-value less than 1.0E-5 against the NR, NT, Swiss-Prot, KEGG, COG, and GO databases (Xu et al., 2018). The Unigenes were obtained for *T. japonicus* antennae, heads, and abdomen, respectively. The confirmed sequences were deposited in the GenBank database with the accession numbers MN923521, MN923522, and MN923523.

### Putative OBP Gene Identification

Using the BLAST homology search method, the critical OBP gene was obtained from the antennal transcriptome of *T. japonicus*. According to the OBP genes of *Telenomus podisi* from the NCBI, homology searching was conducted by BLAST in BioEdit software to identify the candidate OBP genes. Alignment of all the OBP genes with NCBI non-redundant (NR) protein sequence database was conducted to confirm the candidate OBP genes from *T. japonicus*. The Signal IP (version 4.1)^1^ was used to identify signal peptides in the candidate OBPs. A phylogenetic analysis of the OBPs was performed using maximum likelihood trees constructed using MEGA5.1 with 1000 bootstrap replications (Tamura et al., 2011).

### Cloning, Expression and Purification of OPB Proteins

Total RNA was extracted from 50 antennae of female *T. japonicus* using TRIzol. The first-strand total cDNA was obtained from 96.4 ng RNA by the kit of TransScript^®^ RT/RI Enzyme Mix (Trans, TransGen Biotech, Beijing). According to the database of transcriptome, we searched the sequence of the three OBPs and got rid of the signal peptide by the website of SignalIP^1^. The sequence primers of three OBPs were designed by using Primer Premier 5 (PREMIER Biosoft, Palo Alto, CA, United States), and *Nde*I and *Hin*dIII restriction enzyme-cutting sites (underlined) were added in primer as follows. The sequence primers were used as follows: OBP1 forward: 5′-CCGGATCCCTGAAATGTCGCACCGGCAA-3′, OBP1 reverse: 5′CCAAGCTTTTACTTTTTGTAATTACTTATCCAATCGTCA CA-3′, OBP2 forward: 5′-CCGGATCCAAGCGACCGGATTTTA TTGACGATGACAT-3′; OBP2 reverse: 5′-CCCAAGCTTTTA CACGATGAACCACATTTCCGGACAATTATTTATTATACAC TG-3′, OBP3 forward: 5′-CCGGATCCAAACTCTCAGTGCCT CAGCTCAAAGG-3′; OBP3 reverse: 5′-CCCAAGCTTTCATG GAAAGATGTACATTTCTGGATTTTTATGATACCAGCACC-3′. Each PCR reaction consisted of the 1-μl cDNA template, 1 μl forward and reverse primers, 22 μl dd H_2_O, and 25 μl Premix Taq. The PCR program was an initial denaturation at 95°C for 5 min, followed by 35 cycles of 95°C for 30 s, 55°C for 30 s, 72°C for 1 min, and a final extension of 72°C for 10 min. The PCR products were detected by 1.5% agarose gel electrophoresis and were recycled by the kit of TaKaRa Agarose Gel DNA Purification (TaKaRa). Once the PCR products were obtained, the positive clones of the three OBPs were digested with *Nde*I and *Hin*dIII simultaneously with the *pEASY*^®^-T1 Cloning vector and then transformed into Trans-T1 competent cells to amplify. The positive plasmids were extracted by the kit of plasmid mini (TIANGEN, TIANGEN BIOTECH, Beijing), and then the plasmids were transformed into the *Escherichia coli* BL21 competent cells for amplification. The positive clones of the three OBPs were selected for culture to an OD_600_ value of 0.6, and the bacteria solution were divided into two parts, one for control and the other for further experiments. The isopropyl-β-D-thiogalactopyranoside (IPTG) was added at a final concentration of 1 mM, and the protein expression was conducted at 15°C, 220 rpm for 16 h. The commensurable lysate was added to the bacteria solution for ultrasonication (sonicated for 30 min, worked for 3 s, stopped for 6 s). After the crushing, the supernatant and the precipitate were collected by centrifugation (13,000 rpm for 30 min at 4°C). Pilot expression levels of each OBP were detected by SDS-PAGE and Western Blot (Primary antibody: Mouse-anti-His mAb, GenScript, Cat. No. A00186).

The supernatant was loaded to a HisTrap affinity chromatography column (TaKaRa, Dalian, China) and the speed was 1 ml/min. The target proteins were eluted by Buffer 20 (50 mM Tris–Hcl, 150 mM NaCl, 20 mM imidazole, pH 8.0), Buffer 50 (50 mM Tris–Hcl, 150 mM NaCl, 50 mM imidazole, pH 8.0), and Buffer 500 (50 mM Tris–Hcl, 150 mM NaCl, 500 mM imidazole, pH 8.0) separately. Different streaming liquids were collected by the different concentration elutions. The OBP protein sample which has the single band was detected by SDS-PAGE and Western Blot for the protein purity and molecular weight. The protein concentrations were measured by Bradford protein assay with BSA as a standard (TIANGEN, TIANGEN BIOTECH, Beijing). The OBP proteins were then desalted through extensive dialysis, lyophilized, and stored at −80°C until use.

### Competitive Fluorescence-Binding Assay

The fluorescence spectrophotometer (HORIBA FluoroMax^®^-4) was used to measure the emission fluorescence in a right-angle configuration with a 1 cm light path quartz cuvette. The excitation wavelength was 337 nm, and the range of the scanning emission wavelength was 380–450 nm. The protein was dissolved in 50 mM Tris–HCl buffer (PH = 7.4), and all the ligands that are used in the experiments were dissolved in the 1 mM methanol solutions. N-Phenyl-1-naphtylamine (1-NPN) was used as the fluorescent probe, and the binding constants of OBP with 1-NPN were determined according to the Scatchard equation. Then, the binding abilities of *T. japonicus* OBPs and (*E*)-2-decenal were investigated following the protocol as described by Zhong et al. (2018). Each OBP was tested three times and means were used for the analysis.

### Data Analysis

For determination of binding constants, the intensity values corresponding to the maximum fluorescence emission were plotted against the total concentration of 1-NPN (Gong et al., 2009). The binding curve was linearized using the Scatchard equation. The dissociation constant was calculated using the formula K_*d*_ = [IC_50_]/(1 + [1-NPN]/K_1__–__NPN_), where IC_50_ is the (*E*)-2-decenal concentration at which the fluorescence intensity of [OBP/1-NPN] is reduced by half of the highest value, [1-NPN] is the dissociative concentration of 1-NPN, and K_1__–__NPN_ is the dissociation constant of OBP/1-NPN.

## Results

### Overview of *T. japonicus* Transcriptome

A total of 64 290 024, 48 155 024, and 53 983 790 raw reads were obtained from the antenna, abdomen, and head of *T. japonicus*, respectively. After removing the reads with adapter, low-quality reads, and contaminative reads, 63 783 998, 47 369 672, and 53 191 614 valid reads were obtained from the antenna, abdomen, and head of *T. japonicus*, respectively (Supplementary Table S1). 59 214 transcript and 49 673 genes were obtained by *de novo* splicing use Trinity software (Supplementary Table S2). The obtained genes were matched with 5 public databases (Swiss-Prot, NR, KEGG, KOG, and Pfam) with an *E*-value less than 1.0E-10, and functional annotation was done by sequence similarity. The alignment of sequence similarity was searched with the BLAST (Blast Local Alignment Search Tool) algorithm. The result of annotation showed that 21 846, 25 735, 22 663, 9 577, 12 889, and 19 962 genes were annotated by Swiss-Prot, NR, Pfam, KEGG, KOG, and GO, respectively (Supplementary Table S3). A total of 19 962 genes was annotated to 50 classification of Gene Ontology (GO) function and categorized by biological process, cellular component, and molecular function (Supplementary Figure S1). The GO functions more directly related to insect olfaction in the list of biological processes included transmembrane transport, protein transport, and intracellular signal transduction.

Moreover, 9 577 genes were annotated to Kyoto encyclopedia of genes and genomes (KEGG) classification, and categorized by metabolism, organismal systems, cellular processes, genetic information processing, and environmental information processing in descending order of abundance (Supplementary Figure S2). The categories more directly related to insect olfaction in KEGG classification included the sensory system, nervous system, signaling molecules and interaction, signal transduction, membrane transport, and cell communication.

### Identification and Sequence Analysis of TjapOBPs

**FIGURE 1:**
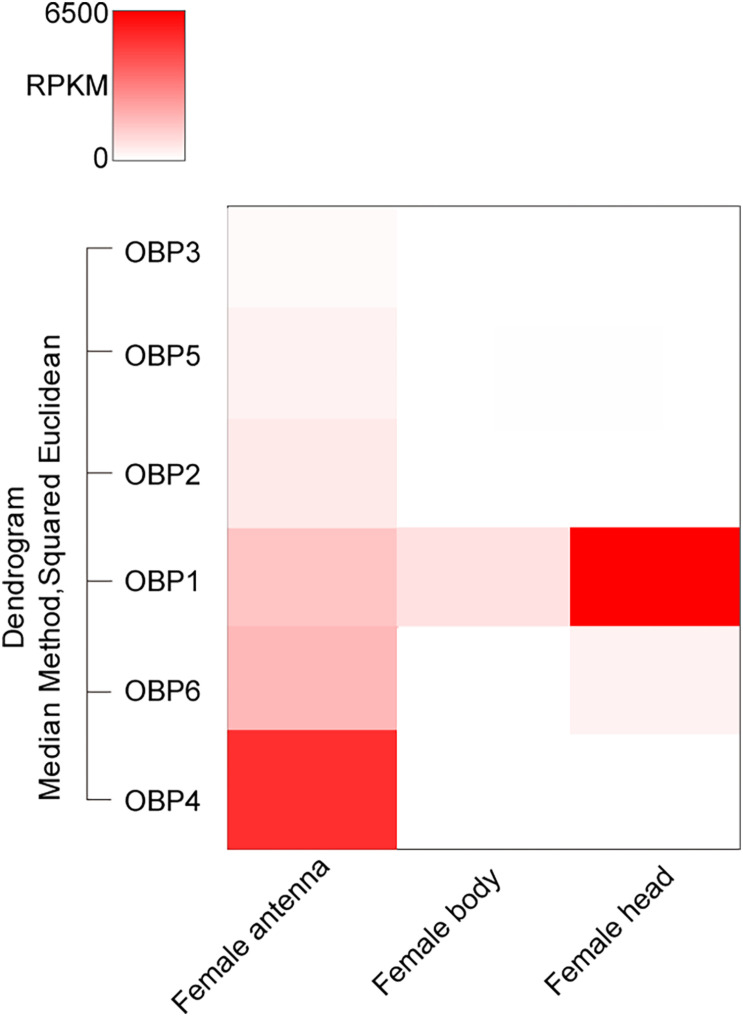
Expression patterns of annotated OBP genes in tested *Trissolcus japonicus* tissues.

**FIGURE 2:**
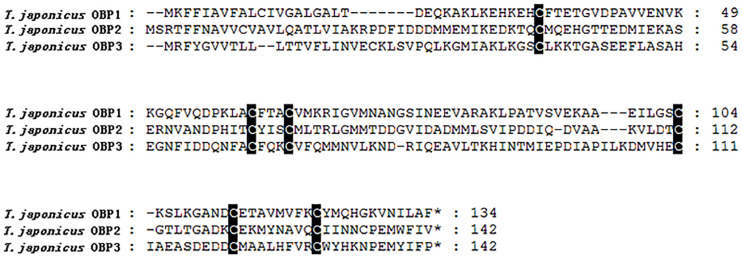
Alignment of amino acid sequences of OBPs in *Trissolcus japonicus*.

**FIGURE 3:**
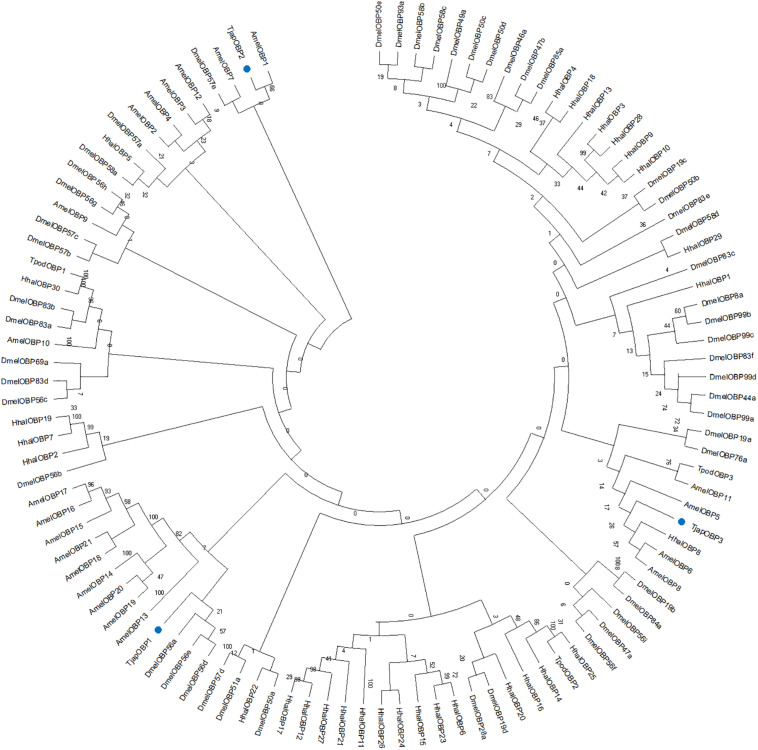
Maximum-likelihood dendrogram based on protein sequences of candidate OBPs from *Trissolcus japonicus* (Tjap), and *Telenomus podis* (Tpod), *Halyomorpha halys* (Hhal), *Drosophila melanogaster* (Dmel), and *Apis mellifera* (Amel). Blue dots indicate TjapOBP1, TjapOBP2, and TjapOBP3.

Totally six *T. japonicus* OBPs (*Tjap*OBPS) were found in the tested tissues of *T. japonicus* (Figure 1 and Supplementary Table S4). *Tjap*OBP1 was relatively abundant among all annotated OBPs and broadly expressed in antenna, abdomen, and head without antenna, whereas *Tjap*OBP2 and *Tjap*OBP3 showed a much lower expression compared with *Tjap*OBP1 and a relatively higher expression in antenna compared with abdomen and head without antenna. Alignment of the amino acid sequences of these three *Tjap*OBPs with full-length transcripts contained six conserved cysteine residues (Figure 2), indicating they were classified as classical OBPs. We compared the selected three *Tjap*OBPs with those of its host *H. haly*s and congeneric species *Telenomus podisi*, as well as other model insects such as the fruit fly *Drosophila melanogaster* and honeybee, *Apis mellifera*. According to the Bayesian Information Criterion (BIC) score, the Whelan and Goldman (WAG) model was used for the phylogenetic analysis of the *Tjap*OBPs. A phylogenetic tree of the *Tjap*OBPs was constructed according to orthologs from *H. halys*, *T. podisi*, *D. melanogaster*, and *A. mellifera* (Figure 3). *Tjap*OBP1, *Tjap*OBP2, and *Tjap*OBP3 were not clearly linked to each other (bootstrap support 0). *Tjap*OBP1 was distantly related to *D. melanogaster* OBP56a, 56e, and 57d (bootstrap support 21). *Tjap*OBP2 was most closely related to *A. mellifera* OBP1 (bootstrap support 66). *Tjap*OBP3 was distantly related to *H. halys* OBP8, *A. mellifera* OBP6, and *A. mellifera* OBP8 (bootstrap support 26).

### Expression and Purification of TjapOBP1, TjapOBP2, and TjapOBP3

**FIGURE 4:**
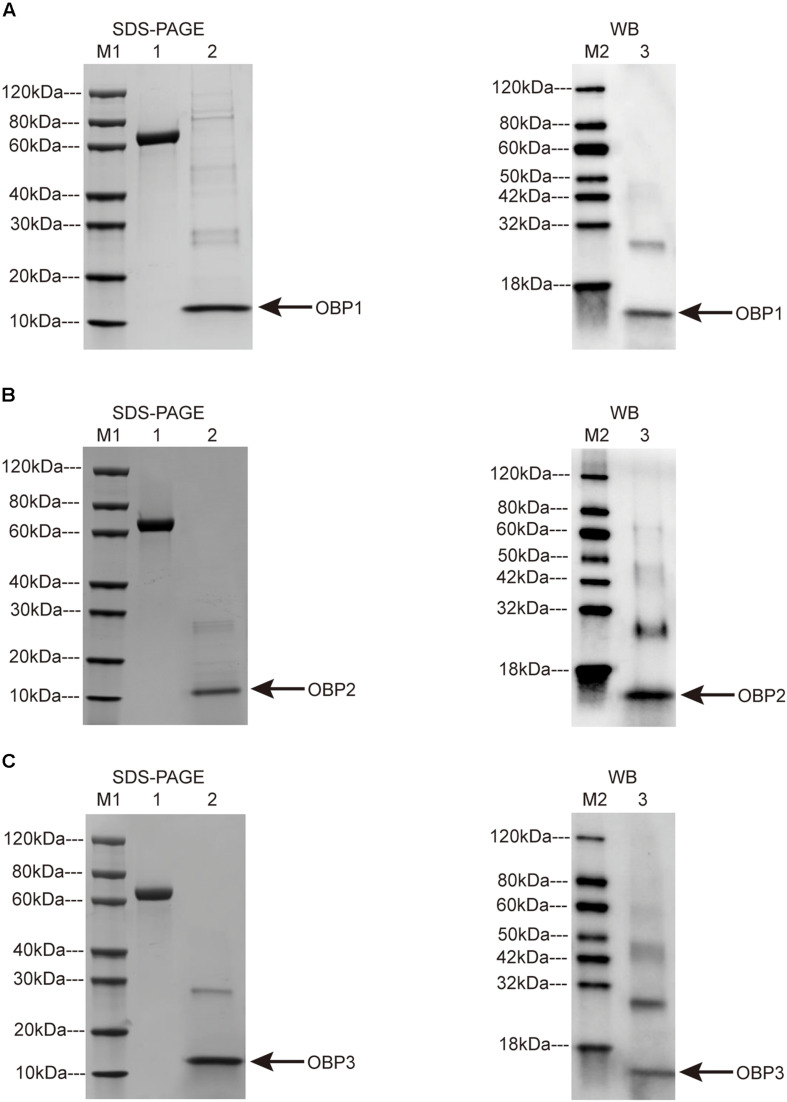
SDS-PAGE (left) and Western blot (right) analysis of *Trissolcus japonicus* OBP1 **(A)**, *T. japonicus* OBP2 **(B)**, and *T. japonicus* OBP3 **(C)**. M1: Protein Marker 1; M2: Protein Marker 2; 1: BSA, 2.0 μg; 2: SDS-PAGE OBPs; 3: Western blot OBPs. Arrows indicate the protein bands.

*Tjap*OBP genes were cloned *in vitro* through a bacterial expression system. After the cleavage of the His-tag and purification, sodium dodecyl sulfate-polyacrylamide gel electrophoresis (SDS-PAGE) and western blotting showed that the molecular masses of *Tjap*OPB1, *Tjap*OBP2, and *Tjap*OBP3 were ∼14, 15, and 15 kDa, respectively. The molecular mass information obtained from SDS-PAGE and western blot tests was consistent with the amino acid lengths predicted from the *T. japonicus* transcriptome (Figure 4). There were great expressions for target proteins when the condition is 15°C, 150 rpm, and 16 h for induction. *Tjap*OBP1 and *Tjap*OBP3 had a large amount of expression in inclusion body after sonication and centrifugation; on the contrary, *Tjap*OBP2 had a large amount of expression in supernatant (Figure 4). The final proteins were obtained by the purification and concentration, and the final concentrations were 1.2, 1.4, and 1.1 mg/ml for *Tjap*OBP1, *Tjap*OBP2, and *Tjap*BP3, respectively.

### Binding Activities of TjapOBP1, TjapOBP2, and TjapOBP3 to (E)-2-Decenal

**FIGURE 5:**
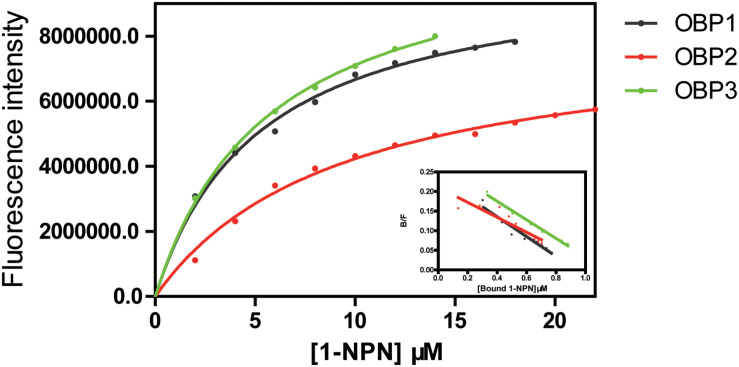
Competitive fluorescence binding of *Trissolcus japonicus* OBPs and 1-NPN.

**FIGURE 6:**
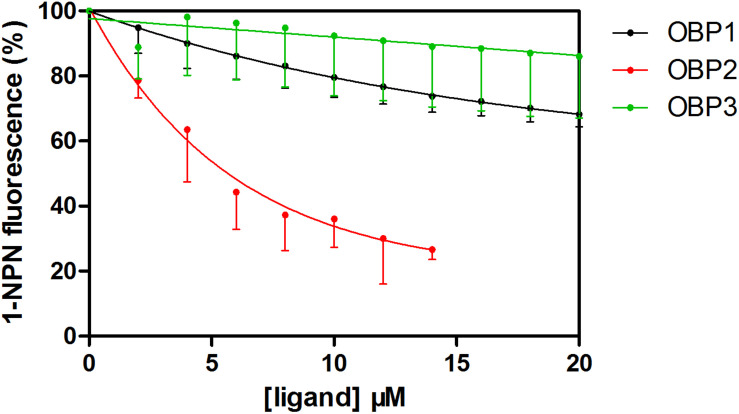
Competitive fluorescence binding of *Trissolcus japonicus* OBPs and (*E*)-2-decenal with 1-NPN.

The fluorescent probe 1-NPN was used to bind the selected OBPs. Significant increases in fluorescence intensity levels were observed when the OBPs were added. Among the tested OBPs, *Tjap*OBP1 revealed the highest slope (Figure 5). We then challenged each OBP/1-NPN complex with (*E*)-2-decenal to test their binding affinities to this alarm pheromone component. All three OBPs bound to (*E*)-2-decenal (Figure 6). *Tjap*OBP2 had a strong ability to bind (*E*)-2-decenal, and the value of kd was 5.68 ± 0.47 μM and the value of IC50 was 7.92 ± 1.03 μM, whereas, *Tjap*OBP1 and *Tjap*OBP3 had a weak ability to bind (*E*)-2-decenal.

## Discussion

The molecular mechanisms of insect olfaction have been widely investigated, particularly since high-throughput sequencing has provided easy access to an increasing amount of genomic and transcriptomic data (Pelosi et al., 2018). OBPs together with olfactory receptors (ORs) are crucial in insect-specific and -sensitive olfaction (Fan et al., 2011). OBPs have been characterized as small globular water-soluble acidic proteins (about 120–150 amino acids) with a signal peptide in the N-terminal region, six cysteine residues (Cys) in conserved positions, and predominant α-helical domains (Tegoni et al., 2004; Farias et al., 2015; Pelosi et al., 2018). Characterized by signature Cys, OBPs have been classified into four subfamilies: classical, Plus-C, Minus-C, and atypical OBPs (Zhou et al., 2004, Zhou et al., 2010b; Xu et al., 2009; Farias et al., 2015; Paula et al., 2016; Pelosi et al., 2018). In our study, for the first time, we identified six *Tjap*OBPs, and three of them were all classical OBPs with 6 Cys. Previously, three full-length classical OBPs were identified and characterized in *T. podisi* (Farias et al., 2015). Classical OBPs was found in the solitary bee, *Osmia cornuta* (Yin et al., 2013), whereas both Minus-C and classical OBPs were found in *A. mellifera* (Forêt and Maleszka, 2006). However, the numbers of OBPs from both *T. japonicus* and *T. podisi* (i.e., 7 OBPs; Farias et al., 2015) were smaller than those obtained from genome analyses of other Hymenoptera species, e.g., 21 from *A. mellifera* (Forêt and Maleszka, 2006), 22 from *Cotesia vestalis* (Nishimura et al., 2012), and 90 from *Nasonia vitripennis* (Vieira et al., 2012). This might be attributed to the independent evolutionary history of the OBP genes in *T. japonicus* or *T. podisi* specialized in recognizing host semiochemcial cues comparing with other identified hymenopteran OBPs (Farias et al., 2015).

OBPs are divergent both between and within species and only share an average of 10–15% of their residues between species compared to CSPs (Pelosi et al., 2006, 2018). The phylogenetic analyses based on the OBP sequences clearly indicated a restricted conservation between various insect orders (Zhou et al., 2010a). However, few exceptions were observed from previous studies (e.g., Gu et al., 2013; Yin et al., 2013). Farias et al. (2015) confirmed very high similarity between the parasitoid *T. podisi* OBPs and its preferred stink bug host *Euschistus heros* (> 80%) while these OBPs were not orthologous to any known hymenopteran OBPs, suggesting that the parasitoid might have evolved independently and converged to the host OBPs. This would provide a possible explanation on molecular mechanism for the host location of *T. podisi* using *E. heros* semiochemical cues (Borges et al., 2011). However, we did not find close phylogenetic relationships between *Tjap*OBPs and its host *H. halys* OBPs, except *Tjap*OBP3 and *H. halys* OBP8. *Tjap*OBP2 was mostly related to *A. mellifera* OBP1, indicating that these highly conserved OBPs would have a similar function in both hymenopteran species, e.g., recognizing similar odors. The three *Tjap*OBPs mapped to different parts of the OBP phylogeny, which might be attributed to different genes for recognition of different odors (Farias et al., 2015). Our competitive fluorescence-binding assay result also showed that the three *Tjap*OBPs had different binding abilities to (*E*)-2-decenal.

Pentatomid stink bugs release a wide variety of hydrocarbons, aldehydes, oxo-aldehydes, and other volatile compounds from the dorsal abdominal glands of nymphs, and the metathoracic glands of adults (Weber et al., 2017). As a major component of the defensive compounds in the stink bugs, (*E*)-2-decenal is emitted by BMSB adults and third-instar nymphs and functions as an alarm pheromone in BMSB (Harris et al., 2015; Zhong et al., 2017, 2018). It also functions as a repellent allomone to *T. japonicus* that significantly extends the host searching time of the parasitoid in the laboratory bioassays (Zhong et al., 2017). This alarm pheromone component was sensed by olfactory reception through *H. halys* OBP25, OBP30, OBP16, OBP8, and OBP4 in BMSB (Zhong et al., 2018; Sun et al., 2020). Interestingly, our results showed that *Tjap*OBP3 was distantly related to *H. halys* OBP8. Thus, it is very possible that *Tjap*OBP3 and *H. halys* OBP8 might evolve from closely related genes and thus are involved in detecting similar odors. The similar OBPs in the parasitoid and its host could suggest that the parasitoid can recognize host semiochemicals (Farias et al., 2015). This was already confirmed in *T. podisi* and its host *E. heros* that the parasitoid used the host sex pheromone to locate its host (Borges et al., 2011). Vandermoten et al. (2011) also found that SaveOBP3 in the aphid *Sitobion avenae* and EbalOBP3 in its predator, the syrphid fly *Episyrphus balteatus*, shared higher similarity and both OBPs are specifically tuned to (*E*)-β-farnesene, acting as an alarm pheromone for aphid and a kairomone for the natural enemies.

Our present study showed that the chemoreception of *T. japonicus* to (*E*)-2-decenal through its olfactory system is slightly complex. The binding ability of *Tjap*OBP2 to the (*E*)-2-decenal is much stronger than *Tjap*OBP1 and *Tjap*OBP3, while the latter two OBPs still have weaker affinities to the volatile compound. We could deduce that *Tjap*OBP2 is involved in a rapid response pathway that drives the initial escape behavior of *T. japonicus* within a short time period when the parasitoid encounters BMSB in the field, while *Tjap*OBP1 and *Tjap*OBP3 would be also involved in this alarm pheromone reception process but not as specific as *Tjap*OBP2. Further studies are still needed to illustrate the complete olfactory mechanism of *T. japonicus* in host location behavior, which will provide a molecular basis in developing behavioral-mediating tools such as gene silencing/RNA interference to enhance the efficacy of biological control of *H. halys* with this important parasitoid.

## Conclusion

We identified three classical OBPs from totally six OBPs in *T. japonicus*, namely, *Tjap*OPB1, *Tjap*OBP2, and *Tjap*OBP3. We also tested their binding functions with (*E*)-2-decenal, an alarm pheromone component of *H. halys*. *Tjap*OBP2 showed the highest binding ability to (*E*)-2-decenal, indicating that this odor carrier protein might play an important role in *T. japonicus* detecting the (*E*)-2-decenal.

## Data Availability Statement

The sequencing data has been deposited into GenBank (accession: MN923521, MN923522, and MN923523).

## Author Contributions

JZ, GC, and FZ contributed to conceptualization. YZ contributed data curation. YZ, KR, and FL contributed to the formal analysis. FZ contributed to the funding acquisition. YZ, JZ, and RT contributed to the investigation. RT and YZ contributed to the methodology. FZ contributed to the project administration. JZ and GC contributed to resources. JZ and FL contributed to the software. YZ and JZ contributed to the writing of the original draft. KR and FZ contributed to the writing – review and editing. All authors contributed to the article and approved the submitted version.

## Conflict of Interest

The authors declare that the research was conducted in the absence of any commercial or financial relationships that could be construed as a potential conflict of interest.

## Footnotes

1 http://www.cbs.dtu.dk/services/SignalP/

## Supplementary Material

TABLE S1 Overview of RNA-sequencing data of *Trissolcus japonicus*.

TABLE S2 Assembly of unigene and transcripts of *Trissolcus japonicus*.

TABLE S3 Annotation of *Trissolcus japonicus* unigenes with BLAST.

TABLE S4 The FPKM values of OBPs in different tissues of *Trissolcus japonicus*.

FIGURE S1 Gene ontology (GO) classification of *T. japonicus* transcriptome. Unigenes were classified into three main categories: biological process, cellular component, and molecular function. The Y-axis indicates the percentage of a specific category of genes in each main category.

FIGURE S2 KEGG functional classification of *T. japonicus* transcriptome.

## References

[B1] Abram P. K. Talamas E. J. Acheampong S. Mason P. G. Gariepy T. D. (2019). First detection of the samurai wasp, *Trissolcus japonicus* (Ashmead) (*Hymenoptera*: *Scelionidae*), in Canada. *J. Hymenop. Res.* 68 29–36. 10.3897/jhr.68.32203

[B2] Borges M. Moraes M. C. B. Peixoto M. F. Pires C. S. S. Sujii E. R. Laumann R. A. (2011). Monitoring the neotropical brown stink bug Euschistus heros (F.) (*Hemiptera*: *Pentaomidae*) with pheromone baited-traps in soybean fields. *J App Entomol* 135 68–80. 10.1111/j.1439-0418.2010.01507.x

[B3] Botch P. S. Delfosse E. S. (2018). Host-acceptance behavior of *Trissolcus japonicus* (*Hymenoptera*: *Scelionidae*) reared on the invasive *Halyomorpha halys* (*Heteroptera*: *Pentatomidae*) and nontarget species. *Environ. Entomol.* 47 403–411. 10.1093/ee/nvy014 29506058

[B4] Bruyne M. D. Baker T. C. (2008). Odor detection in insects: volatile codes. *J. Chem. Ecol.* 34 882–897. 10.1007/s10886-008-9485-4 18535862

[B5] Calvello M. Gueera N. Brandazza A. Dambrossio C. Scaloni A. Dani F. R. (2003). Soluble proteins of chemical communication in the social wasp *Polistes dominulus*. *Cell Mol Life Sci.* 60 1933–1943. 10.1007/s00018-003-3186-5 14523553 PMC11138633

[B6] Conchou L. Lucas P. Meslin C. Proffit M. Staudt M. Renou M. (2019). Insect odorscapes: from plant volatiles to natural olfactory scenes. *Front. Physiol.* 10:972. 10.3389/fphys.2019.00972 31427985 PMC6688386

[B7] Fan J. Francis F. Liu Y. Chen J. L. Cheng D. F. (2011). An overview of odorant binding protein functions in insect peripheral olfactory reception. *Genet. Mol. Res.* 10 3056–3069. 10.4238/2011.december.8.2 22180039

[B8] Farias L. R. Schimmelpfeng P. H. C. Togawa R. C. Costa M. M. C. Grynberg P. Martins N. F. (2015). Transcriptome-based identification of highly similar odorant-binding proteins among neotropical stink bugs and their egg parasitoid. *PLoS One* 10:e0132286. 10.1371/journal.pone.0132286 26161752 PMC4498631

[B9] Forêt S. Maleszka R. (2006). Function and evolution of a gene family encoding odorant binding-like proteins in a social insect, the honey bee (*Apis mellifera*). *Genome Res.* 16 1404–1413. 10.1101/gr.5075706 17065610 PMC1626642

[B10] Gong Z.-J. Zhou W.-W. Yu H.-Z. Mao C.-G. Zhang C.-X. Cheng J. A. (2009). Cloning, expression and functional analysis of a general odorant-binding protein 2 gene of the rice striped stem borer, Chilo suppressalis (Walker) (*Lepidoptera*: *Pyralidae*). *Insect Mol. Biol.* 18 405–417. 10.1111/j.1365-2583.2009.00886.x 19523072

[B11] Gu S.-H. Wu K.-M. Guo Y.-Y. Field L. M. Pickett J. A. Zhang Y.-J. (2013). Identification and expression profiling of odorant binding proteins and chemosensory proteins between two wingless morphs and a winged morph of the cotton aphid *Aphis gossypii* Glover. *PLoS One* 8:e73524. 10.1371/journal.pone.0073524 24073197 PMC3779235

[B12] Haas B. J. Papanicolaou A. Yassour M. Grabherr M. Blood P. D. Bowden J. (2013). *De novo* transcript sequence reconstruction from RNA-seq using the Trinity platform for reference generation and analysis. *Nat. Protoc.* 8 1494–1512. 10.1038/nprot.2013.084 23845962 PMC3875132

[B13] Harris C. Abubeker S. Yu M. Leskey T. Zhang A. (2015). Semiochemical production and laboratory behavior response of the brown marmorated stink bug *Halyomorpha halys*. *PLoS One* 10:e0140876. 10.1371/journal.pone.0140876 26528717 PMC4631522

[B14] Haye T. Gariepy T. D. Hoelmer K. Rossi J. P. Streito J. C. Tassus T. (2015). Range expansion of the invasive brown marmorated stinkbug, *Halyomorpha halys*: an increasing threat to field, fruit and vegetable crops worldwide. *J. Pest Sci.* 88 665–673. 10.1007/s10340-015-0670-2

[B15] Haye T. Moraglio S. T. Stahl J. Visentin S. Gregorio T. Tavella L. (2019). Fundamental host range of *Trissolcus japonicus* in Europe. *J. Pest Sci.* 92 1–12.

[B16] Haye T. Weber D. C. (2017). Special issue on the brown marmorated stink bug, *Halyomorpha halys*: an emerging pest of global concern. *J. Pest Sci.* 90 987–988. 10.1007/s10340-017-0897-1

[B17] Hedstrom C. Lowenstein D. Andrews H. Bai B. Wiman N. (2017). Pentatomid host suitability and the discovery of introduced populations of *Trissolcus japonicus* in Oregon. *J. Pest Sci.* 90 1169–1179. 10.1007/s10340-017-0892-6

[B18] Herlihy M. V. Talamas E. J. Weber D. B. (2016). Attack and success of native and exotic parasitoids on eggs of *Halyomorpha halys* in three Maryland habitats. *PLoS One* 11:e0150275. 10.1371/journal.pone.0150275 26983012 PMC4794195

[B19] Hoebeke E. R. Carter M. E. (2003). *Halyomorpha halys* (Stål) (*Heteroptera*: *Pentatomidae*): a polyphagous plant pest from Asia newly detected in North America. *Proc. Entomol. Soc. Wash.* 105 225–237.

[B20] Leal W. S. (2005). Pheromone reception. *Top. Curr. Chem.* 240 1–36.

[B21] Leal W. S. (2013). Odorant reception in insects: roles of receptors, binding proteins, and degrading enzymes. *Annu. Rev. Entomol.* 58 373–391. 10.1146/annurev-ento-120811-153635 23020622

[B22] Lee D. Short B. D. Joseph S. V. Christopher J. Leskey T. C. Bergh J. C. (2013). Review of the biology, ecology, and management of *Halyomorpha halys* (*Hemiptera*: *Pentatomidae*) in China, Japan, and the Republic of Korea. *Environ. Entomol.* 42 627–641. 23905725 10.1603/EN13006

[B23] Leong W. H. Teh S. Y. Hossain M. M. Nadarajaw T. Zabidi-Hussin Z. Chin W. Y. (2020). Application, monitoring and adverse effect in pesticide use: the importance of reinforcement of Good Agricultural Practices (GAPs). *J. Environ. Manag.* 260:109987. 10.1016/j.jenvman.2019.109987 32090796

[B24] Leskey T. C. Nielsen A. L. (2018). Impact of the invasion brown marmorated stink bug in North America and Europe: history, biology, ecology, and management. *Ann. Rev. Entomol.* 63 599–618. 10.1146/annurev-ento-020117-043226 29068708

[B25] Leskey T. C. Short B. D. Butler B. R. Wright S. E. (2012). Impact of the invasive brown marmorated stink bug, *Halyomorpha halys* (Stål), in mid-Atlantic tree fruit orchards in the United States: case studies of commercial management. *Psyche* 2012 1–14. 10.1155/2012/535062

[B26] Li F. Li D. Dewer Y. Qu C. Yang Z. Tian J. (2019). Discrimination of oviposition deterrent volatile -ionone by odorant-binding proteins 1 and 4 in the whitefly bemisia tabaci. *Biomolecules* 9:563. 10.3390/biom9100563 31623354 PMC6843521

[B27] Li K. Yang X. Xu G. Cao Y. Lu B. Peng Z. (2015). Identification of putative odorant binding protein genes in *Asecodes hispinarum*, a parasitoid of coconut leaf beetle (*Brontispa longissima*) by antennal RNA-Seq analysis. *Biochem. Biophys. Res. Commun* 467 514–520. 10.1016/j.bbrc.2015.10.008 26454175

[B28] McKenzie S. K. Oxley P. R. Kronauer D. J. C. (2014). Comparative genomics and transcriptomics in ants provide new insights into the evolution and function of odorant binding and chemosensory proteins. *BMC Genomics* 15:718. 10.1186/1471-2164-15-718 25159315 PMC4161878

[B29] Milnes J. Wiman N. G. Talamas E. J. Brunner J. F. Hoelmer K. Buffington M. L. (2016). Discovery of an exotic egg parasitoid of the brown marmorated stink bug, *Halyomorpha halys* (Stål) in the Pacific Northwest. *Proc. Entomol. Soc. Wash.* 118 466–470. 10.4289/0013-8797.118.3.466

[B30] Milnes J. M. Beers E. H. (2019). *Trissolcus japonicus* (*Hymenoptera*: *Scelionidae*) causes low levels of parasitism in three North American Pentatomids under field conditions. *J. Insect Sci.* 19 1–6. 10.1093/jisesa/iez074 PMC6687051 31393980

[B31] Nishimura O. Brillada C. Yazawa S. Maffei M. E. Arimura G.-I. (2012). Transcriptome pyrosequencing of the parasitoid wasp *Cotesia vestalis*: genes involved in the antennal odorant-sensory system. *PLoS One* 7:e50664. 10.1371/journal.pone.0050664 23226348 PMC3511342

[B32] Paula D. P. Togawa R. C. Costa M. M. Grynberg R. Martins N. F. Andow D. A. (2016). Identification and expression profile of odorant-binding proteins in *Halyomorha halys* (*Hemiptera*: *Pentatomidae*). *Insect Mol. Biol.* 25 580–594. 10.1111/imb.12243 27170546

[B33] Pelosi P. Iovinella I. Zhu J. Wang G. Dani F. R. (2018). Beyond chemoreception: diverse tasks of soluble olfactory proteins in insects. *Biol. Rev.* 93 184–200. 10.1111/brv.12339 28480618

[B34] Pelosi P. Zhou J. J. Ban L. P. Calvello M. (2006). Soluble proteins in insect chemical communication. *Cell Mol Life Sci.* 63 1658–1676. 10.1007/s00018-005-5607-0 16786224 PMC11136032

[B35] Qin W. L. (1990). The regularity outbreak and control technique of *Halyomorpha picus* Fabricius. *Plant Prot* 16 22–23.

[B36] Sabbatini Peverieri G. Talamas E. Bon M. C. Marianelli L. Bernardinelli I. Malossini G. (2018). Two Asian egg parasitoids of *Halyomorpha halys* (Stål) (*Hemiptera*, *Pentatomidae*) emerge in northern Italy: *Trissolcus mitsukurii* (Ashmead) and *Trissolcus japonicus* (Ashmead) (*Hymenoptera*, *Scelionidae*). *J. Hymenop. Res.* 37 57–63.

[B37] Schneider D. (1969). Insect olfaction: deciphering system for chemical messages. *Science* 163 1031–1037. 10.1126/science.163.3871.1031 4885069

[B38] Stahl J. Tortoici F. Pontini M. Bon M.-C. Hoelmer K. Marazzi C. (2018). First discovery of adventive populations of *Trissolcus japonicus* in Europe. *J. Pest Sci.* 92 371–379. 10.1007/s10340-018-1061-2

[B39] Steinbrecht R. A. (1998). Odorant-binding proteins: expression and function. *Ann. N. Y. Acad. Sci.* 855 323–332. 10.1111/j.1749-6632.1998.tb10591.x 10049226

[B40] Sun D. D. Huang Y. Qin Z. J. Zhan H. X. Zhang J. P. Liu Y. (2020). Identification of candidate olfactory genes in the antennal transcriptome of the stink bug *Halyomorpha halys. Front. Psychol.* 11:876. 10.3389/fphys.2020.00876 32792985 PMC7394822

[B41] Talamas E. J. Buffington M. Hoelmer K. (2013). New synonymy of *Trissolcus halyomorphae* Yang. *J. Hymenopt. Res.* 33 113–117. 10.3897/jhr.33.5627

[B42] Talamas E. J. Herlihy M. V. Dieckhoff C. Hoelmer K. A. Buffingtion M. L. Bon M. C. (2015). *Trissolcus japonicus* (Ashmead) (*Hymenoptera*, *Scelionidae*) emerges in North America. *J. Hymenopt. Res.* 43 119–128.

[B43] Tamura K. Peterson D. Peterson N. Stecher G. Nei M. Kumar S. (2011). MEGA5: molecular evolutionary genetics analysis using maximum likelihood, evolutionary distance, and maximum parsimony methods. *Mol. Biol. Evol.* 28 2731–2739. 10.1093/molbev/msr121 21546353 PMC3203626

[B44] Tegoni M. Campanacci V. Cambillau C. (2004). Structural aspects of sexual attraction and chemical communication in insects. *Trends Biochem Sci.* 29 257–264. 10.1016/j.tibs.2004.03.003 15130562

[B45] Valentine R. E. Nielsen A. L. Wiman N. G. Lee D. H. Fonseca D. M. (2017). Global invasion network of the brown marmorated stink bug. *Halyomorpha halys*. *Sci. Rep.* 7:9866. 10.1038/s41598-017-10315-z PMC5575200 28852110

[B46] Vandermoten S. Francis F. Haubruge E. Leal W. S. (2011). Conserved odorant-binding proteins from aphids and eavesdropping predators. *PLoS One* 6:e23608. 10.1371/journal.pone.0023608 21912599 PMC3160308

[B47] Vieira F. G. Forêt S. He X. Rozas J. Field L. M. Zhou J. J. (2012). Unique features of odorant-binding proteins of the parasitoid wasp *Nasonia vitripennis* revealed by genome annotation and comparative analyses. *PLoS One* 7:e43034. 10.1371/journal.pone.0043034 22952629 PMC3428353

[B48] Vogt R. G. Riddiford L. M. (1981). Pheromone binding and inactivation by moth antenna. *Nature* 5828 161–163. 10.1038/293161a0 18074618

[B49] Weber D. C. Morrison W. R. Khrimian A. Rice K. B. Leskey T. C. Rodriguez-Saona C. (2017). Chemical ecology of *Halyomorpha halys*: discoveries and applications. *J. Pest. Sci.* 90 989–1008. 10.1007/s10340-017-0876-6

[B50] Xu L. T. Zhang Y. Q. Zhang S. H. Deng J. D. Lu M. Zhang L. W. (2018). Comparative analysis of the immune system of an invasive bark beetle, *Dendroctonus valens*, infected by an entomopathogenic fungus. *Dev. Comp. Immunol.* 88 65–69. 10.1016/j.dci.2018.07.002 30017857

[B51] Xu Y. L. He P. Zhang L. Fang S. Q. Dong S. L. Zhang Y. J. (2009). Large-scale identification of odorant-binding proteins and chemosensory proteins from expressed sequence tags in insects. *BMC Genomics* 10:632. 10.1186/1471-2164-10-632 20034407 PMC2808328

[B52] Yang Z. Q. Yao Y. X. Qiu L. F. Li Z. X. (2009). A new species of *Trissolcus* (*Hymenoptera*: *Scelionidae*) parasitizing eggs of *Halyomorpha halys* (*Heteroptera*: *Pentatomidae*) in China with comments on its biology. *Ann. Entomol. Soc. Am.* 102 39–47.

[B53] Yin X.-W. Iovinella I. Marangoni R. Cattonaro F. Flamini G. Sagona S. (2013). Odorant-binding proteins and olfactory coding in the solitary bee *Osmia cornuta*. *Cell Mol. Life Sci.* 70 3029–3039. 10.1007/s00018-013-1308-2 23512006 PMC11113457

[B54] Zhang J. P. Zhang F. Gariepy T. Mason P. Gillespie D. Talamas E. (2017). Seasonal parasitism and host specificity of *Trissolcus japonicus* in northern China. *J. Pest. Sci.* 90 1127–1141. 10.1007/s10340-017-0863-y 28824354 PMC5544787

[B55] Zhong Y. Z. Tang R. Zhang J. P. Yang S. Y. Chen G. H. He K. L. (2018). Behavioral evidence and olfactory reception of a single alarm pheromone component in *Halyomorpha halys*. *Front. Physiol.* 9:1610. 10.3389/fphys.2018.01610 30483157 PMC6243750

[B56] Zhong Y. Z. Zhang J. P. Ren L. L. Tang R. Zhan H. X. Chen G. H. (2017). Behavioral responses of the egg parasitoid *Trissolcus japonicus* to volatiles from adults of its stink bug host *Halyomorpha halys*. *J. Pest Sci.* 90 1097–1105. 10.1007/s10340-017-0884-6

[B57] Zhou C. X. Min S. F. Tang Y. L. Wang M. Q. (2015). Analysis of antennal transcriptome and odorant binding protein expression profiles of the recently identified parasitoid wasp, *Sclerodermus* sp. *Comp. Biochem. Physiol. Part D* 16 10–19. 10.1016/j.cbd.2015.06.003 26164593

[B58] Zhou J. J. Field L. M. He X. L. (2010a). Insect odorant-binding proteins: do they offer an alternative pest control strategy? *Outlooks Pest Manag.* 21 31–34. 10.1564/21feb08 16562962

[B59] Zhou J. J. Vieira F. G. He X. L. Smadja C. Liu R. Rozas J. (2010b). Genome annotation and comparative analyses of the odorant-binding proteins and chemosensory proteins in the pea aphid *Acyrthosiphon pisum*. *Insect Mol. Biol*. 19(Suppl. 2), 113–122. 10.1111/j.1365-2583.2009.00919.x 20482644

[B60] Zhou J. J. Huang W. Zhang G. A. Pickett J. A. Field L. M. (2004). “Plus-C” odorant-binding protein genes in two *Drosophila* species and the malaria mosquito *Anopheles gambiae*. *Gene* 327 117–129. 10.1016/j.gene.2003.11.007 14960367

