# Supplementary material for: Olfactory Reception of Host Alarm Pheromone Component by the Odorant-Binding Proteins in the Samurai Wasp, Trissolcus japonicus (Hymenoptera: Scelionidae)
Source: Front Physiol. 2020 Sep 3;11:1058. doi: 10.3389/fphys.2020.01058 (PMC7494974; doi:10.3389/fphys.2020.01058)
Supplement: TABLE S1 — Overview of RNA-sequencing data of Trissolcus japonicus. [file Table_1.docx]

| Sample | Raw data read | Base | Valid data read | Base | Valid% | Q20% | Q30% | GC% |
| --- | --- | --- | --- | --- | --- | --- | --- | --- |
| Antennae | 64290024 | 9.71G | 63783998 | 9.49G | 99.21 | 99.02 | 96.92 | 46.52 |
| Abdomen | 48155024 | 7.27G | 47369672 | 6.98G | 98.37 | 98.22 | 94.97 | 45.86 |
| Head | 53983790 | 8.15G | 53191614 | 7.85G | 98.53 | 98.36 | 95.3 | 45.13 |

Table S1: Overview of RNA-Sequencing data of *Trissolcus japonicus*

Table S2: Assembly of Unigene and Transcripts of *Trissolcus* *japonicus*

|  | All | Median GC% | Mean GC% | Min length | Median length | Mean length | Max length | Total assembled bases | N50 |
| --- | --- | --- | --- | --- | --- | --- | --- | --- | --- |
| Gene | 49673 | 43.3 | 43.44 | 201 | 409 | 760 | 13115 | 37753302 | 1288 |
| Transcript | 59214 | 43.2 | 43.36 | 201 | 482 | 950 | 13115 | 56303831 | 1804 |

Table S3: Annotation of *Trissolcus japonicus* unigenes with BLAST

| Gene number | Swiss-prot | NR | Pfam | KEGG | KOG |
| --- | --- | --- | --- | --- | --- |
| 49673 | 21846 | 25735 | 22663 | 9577 | 12889 |
| 100% | 43.98% | 51.81% | 45.62% | 19.28% | 25.95% |

Table S4: The FPKM values of OBPs in different tissues of *Trissolcus japonicus*

|  |  | FPKM values |  |
| --- | --- | --- | --- |
| OBPs | Antenna | Abdomen | Head without antenna |
| *Tjap*OBP1 | 1509.80 | 760.95 | 6452.09 |
| *Tjap*OBP2 | 543.43 | 0.00 | 2.13 |
| *Tjap*OBP3 | 148.26 | 0.47 | 1.19 |
| *Tjap*OBP4 | 5274.53 | 1.78 | 29.55 |
| *Tjap*OBP5 | 334.19 | 2.23 | 12.06 |
| *Tjap*OBP6 | 1815.05 | 41.68 | 333.02 |
