# Supplementary figures and images for: Olfactory Reception of Host Alarm Pheromone Component by the Odorant-Binding Proteins in the Samurai Wasp, Trissolcus japonicus (Hymenoptera: Scelionidae)
Source: Front Physiol. 2020 Sep 3;11:1058. doi: 10.3389/fphys.2020.01058 (PMC7494974; doi:10.3389/fphys.2020.01058)

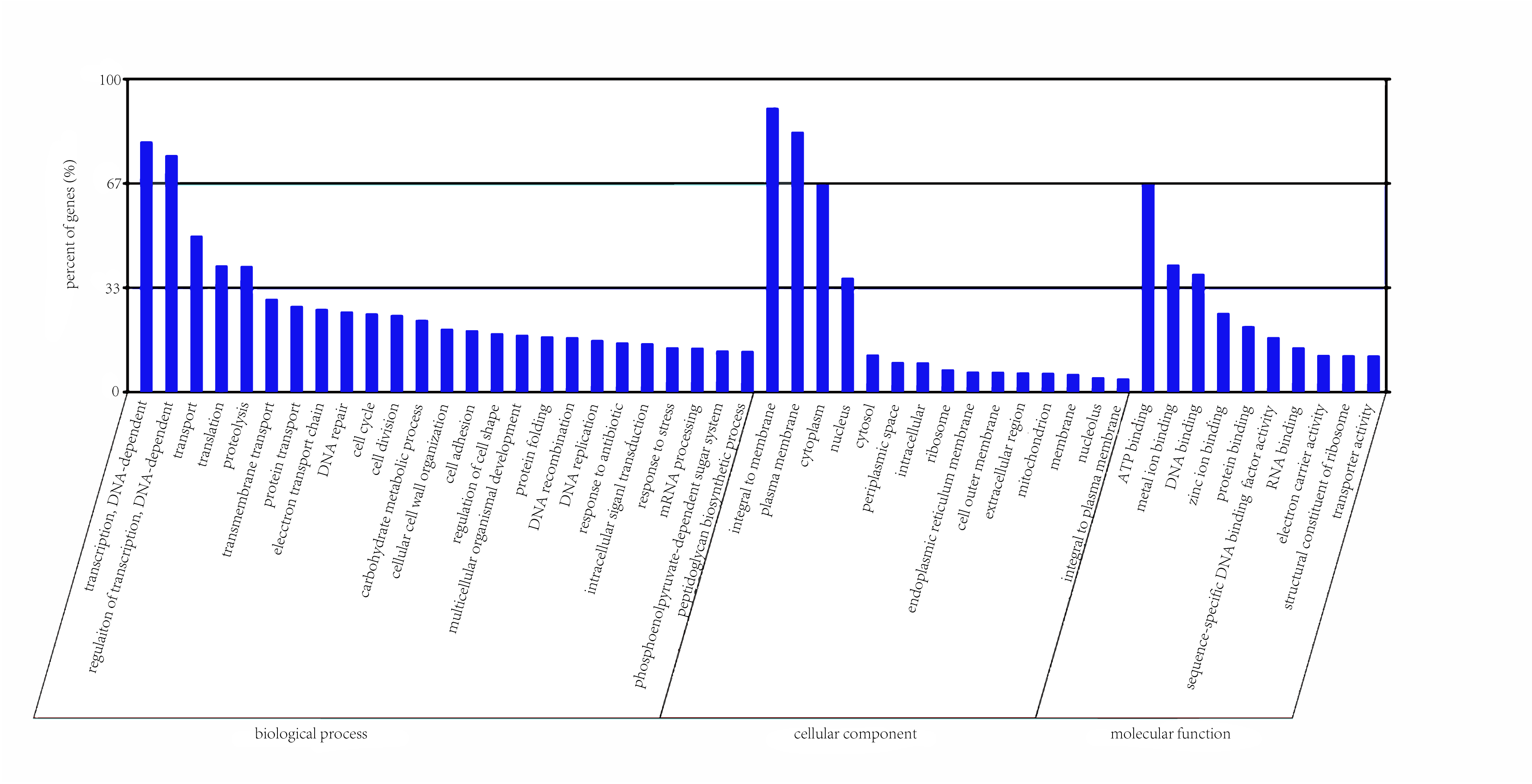

Supplement: FIGURE S1 — Gene ontology (GO) classification of T. japonicus transcriptome. Unigenes were classified into three main categories: biological process, cellular component, and molecular function. The Y-axis indicates the percentage of a specific category of genes in each main category. [file Image_1.TIF]

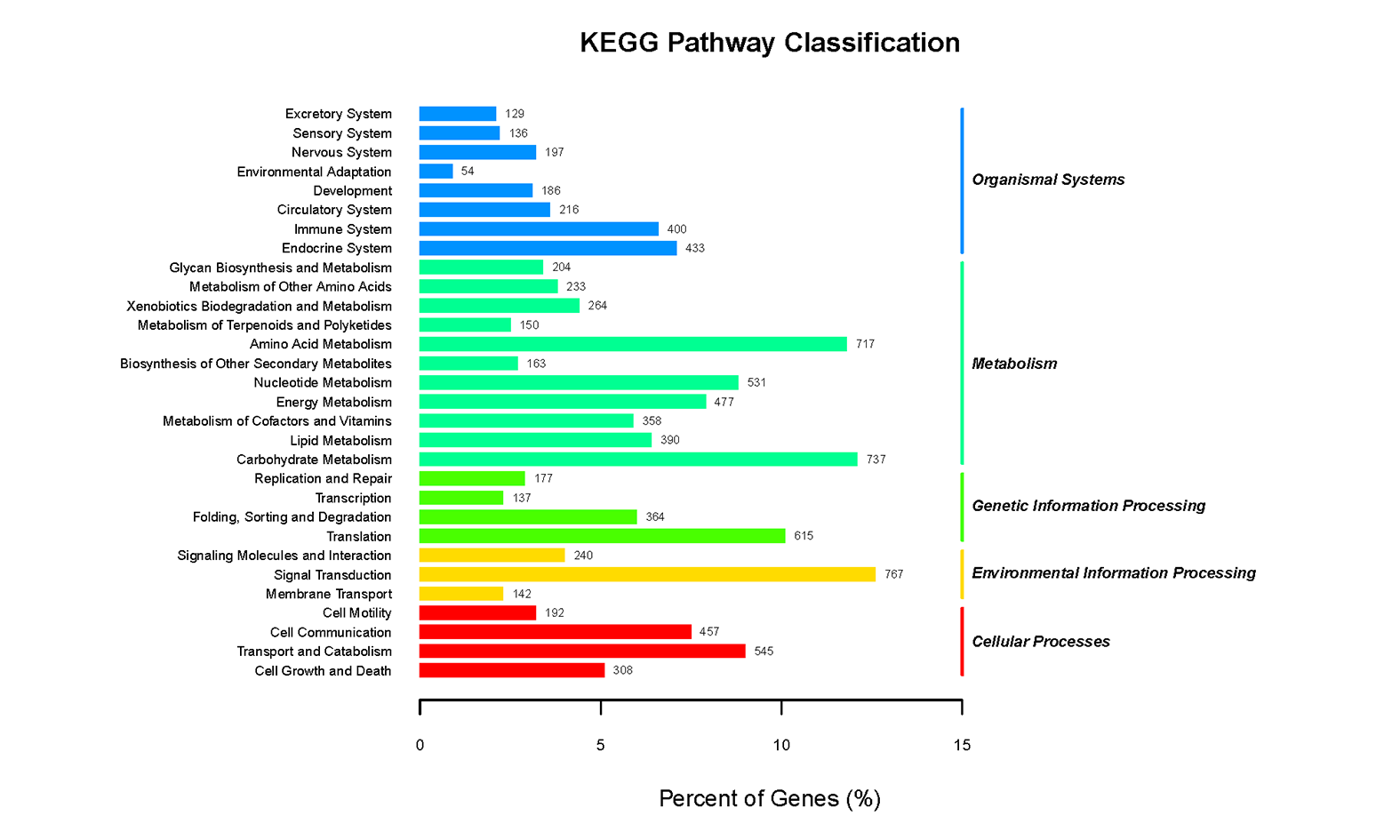

Supplement: FIGURE S2 — KEGG functional classification of T. japonicus transcriptome. [file Image_2.TIF]
