# Supplementary material for: Olfactory Reception of Host Alarm Pheromone Component by the Odorant-Binding Proteins in the Samurai Wasp, Trissolcus japonicus (Hymenoptera: Scelionidae)
Source: Front Physiol. 2020 Sep 3;11:1058. doi: 10.3389/fphys.2020.01058 (PMC7494974; doi:10.3389/fphys.2020.01058)
Supplement: Supplementary file 4 [file Data_Sheet_1.docx]

**Supplementary data**

[**Nucleotide**](C:/Users/hp/AppData/Local/youdao/dict/Application/8.9.3.0/resultui/html/index.html#/javascript:;) [**sequence**](C:/Users/hp/AppData/Local/youdao/dict/Application/8.9.3.0/resultui/html/index.html#/javascript:;)**s of *Trissolcus japonicus* OBPs**

***Tjap*OBP1**

ATGAAATTTTTCATCGCAGTCTTCGCTCTTTGCATCGTTGGAGCTCTTGGTGCTCTGACCGATGAACAAAAAGCCAAACTCAAAGAACACAAAGAACACTGCTTCACTGAGACCGGTGTCGATCCAGCTGTCGTTGAAAATGTCAAGAAAGGCCAATTTGTTCAGGACCCCAAACTTGCTTGCTTCACCGCCTGCGTGATGAAGAGAATTGGAGTTATGAACGCTAATGGATCTATCAACGAAGAAGTTGCCCGTGCTAAACTGCCCGCAACTGTTTCCGTCGAGAAGGCTGCAGAAATTTTGGGCTCTTGCAAATCGCTCAAGGGAGCTAACGACTGTGAAACCGCCGTCATGGTATTCAAGTGCTACATGCAACATGGAAAAGTCAACATCCTGGCTTTCTAA

***Tjap*OBP2**

ATGTCGCGCACATTTTTCAACGCGGTCGTCTGTGTTGCTGTATTACAGGCCACCCTTGTTATTGCTAAGCGACCGGATTTTATTGACGATGACATGATGGAAATGATAAAGGAGGACAAAACTCAGTGCATGCAGGAGCACGGCACAACAGAAGATATGATTGAAAAAGCTAGCGAGAGAAACGTTGCAAACGATCCTCACATTACCTGTTACATCAGTTGTATGTTAACCCGACTCGGAATGATGACTGATGATGGTGTCATTGACGCCGATATGATGTTGAGTGTTATACCAGACGACATTCAAGACGTCGCAGCAAAAGTTTTGGATACATGTGGAACGCTAACCGGTGCAGACAAATGTGAAAAAATGTACAATGCTGTTCAGTGTATAATAAATAATTGTCCGGAAATGTGGTTCATCGTGTAA

***Tjap*OBP3**

ATGAGATTCTACGGTGTTGTAACATTGCTTCTCACTACAGTATTTTTAATCAATGTTGAATGTAAACTCTCAGTGCCTCAGCTCAAAGGTATGATTGCAAAACTCAAAGGATCGTGTTTGAAAAAAACTGGTGCTTCTGAAGAATTTTTAGCGAGTGCACATGAAGGAAATTTCATCGATGATCAAAATTTTGCTTGCTTTCAAAAATGTGTATTTCAAATGATGAATGTATTAAAAAATGACAGAATACAAGAAGCAGTTTTAACTAAACACATCAACACTATGATAGAGCCGGATATAGCTCCTATCTTAAAAGACATGGTGCACGAATGTATTGCTGAAGCCTCCGACGAAGATGATTGTATGGCAGCTCTACATTTCGTTAGGTGCTGGTATCATAAAAATCCAGAAATGTACATCTTTCCATGAA

***Tjap*OBP4**

ATGAAAGCCATCCATATAGCTTTTATTGTTATCGTTGCATTTGCAAGCAACGCATATGGCCAACAAAAAGTCGCGGCCCTTATTGAAAAAGTTGCAAAGAATTGCATGAAGAAAACGGGTGTAAATCGAGATCAAATCATTGAATGTACGATGACACCAACTGACGAATGGGATAGTAATCCTGACTGTACACCTGCAGCCCAATGCTTCTTTGGATGTCCATTCAGTATGCTGCTTGACTCGAAAAAATGCTTCGACTGTGATAAGGGAATAAAAATGATCCAGACCTTCATGAAAGGTCCTGATCTAGAAGAGATGGGTGCCTGCCTTACGAAAGGAATGAAACAGTGTTGCCCAGGACTTCAAAAGGTAGATTGCTGCAAAAATCACTACGACTTAATCGCCTGCATGTACCCAATTTGTGGAGAAATCATCGCTAAACATTTGACTGGCGTGGCAAAATAA

***Tjap*OBP5**

ATGAACAAGTTCTGTCTGTTCGTTCTCTGTTACTCAATCCTGTTCTGGAACATTGCGAGTGCGCTGAAATGTCGCACCGGCAACGAGGAGGACAACGAGCAGTATTACAAAATCATGGACGACTGCAAGAAACGGTACACGATGCAGGACAGCAGAGATTCTGACAGCGACGAATCCATGAGCTCCAACGATGACAACTTCGATCGAAGGTCCATGTCCAGCGGAAGATTCAATGATGATTTCGGAAGAAATTGGATGAACGGGATGAGAAACCAAAATGGTCAAAGGGGAAGAGACTGGAACAACAGGTCCTTATCAGATATCTACAGCCGGAATATGAACCGAAGAATGGGGATGGGAAGAGACTACGATGACCAGCGCTATAACAGACGAAATCAAAACTGGCATTTCACGAATGGAGGAAATTTTGACCGCAGATTTGACGACCAGCACAATCGCGGTTACGACCAGCAGGATCAATCTTGCGCGACACAGTGTTTCTTTAACGAATTGAATCTGGTGGATCAAAGAGGTTTCCCTGACCGTTCGGCAGTGAATTCAATACTGCTGGAAGATATTCAAGATCCCGAGTTGAAAGACTTCGTAGAAGAGGCGGTAGTTCAATGCTTTCATTATCTCAACTTAGAGGGAAAGCAAAACAAGTGTCAGTACTCGCAGCACTTGCTGTCGTGTTTGATAGAAAAAGGCGAAGAGAGATGTGACGATTGGATAAGTAATTACAAAAAGTAA

***Tjap*OBP6**

CGGAACCCGGTTGAGCCGGTCTGTCCACCGGAGTCTGGCAAGTGGCGCAGCGTCGGTGGGCCAGGTAGAGCGGATGAGATTACGTGCGAGTGCGAAGCCGGGCAGCTACGTTGATCGCGCGGTTCTGAGGGCGCGCGCTGTTAAAGAAACGAGACGTTGCGTGGGACGGACCAGTGGGAGAGAAAGAAAGAGAGAGAGAGAGAGAGGGCTGGCTGAAT(sequence incomplete)
